# Missed Opportunities: The Need to Promote Public Knowledge and Awareness of Sugar-Sweetened Beverage Taxes

**DOI:** 10.3390/ijerph18094607

**Published:** 2021-04-27

**Authors:** Emily A. Altman, Kristine A. Madsen, Laura A. Schmidt

**Affiliations:** 1 School of Public Health, University of California, Berkeley, CA 94720, USA; madsenk@berkeley.edu; 2 Philip R. Lee Institute for Health Policy Studies, Department of Humanities and Social Sciences, School of Medicine, University of California at San Francisco, San Francisco, CA 94118, USA; Laura.Schmidt@ucsf.edu

**Keywords:** health policy, nutrition/food, health behavior

## Abstract

Despite a growing body of evidence showing that sugar-sweetened beverage (SSB) taxes nudge consumers away from SSBs, we lack an understanding of people’s awareness and perceptions of SSB taxes and whether tax awareness and perceptions differ based on sociodemographic characteristics. We used serial cross-sectional study intercept surveys (*n* = 2715) in demographically diverse neighborhoods of Berkeley and Oakland in 2015 and 2017, and San Francisco and Richmond in 2017. In the year following successful SSB tax ballot measures, 45% of respondents correctly recalled that an SSB tax had passed in their city. In untaxed cities, 14% of respondents incorrectly thought that a tax had passed. Perceived benefits of SSB taxes to the community and to children’s health were moderate and, like correct recall of an SSB tax, were higher among respondents with higher education levels. Awareness of SSB taxes was low overall, and perceptions about taxes’ benefits varied by educational attainment, reflecting a missed opportunity to educate citizens about how SSB taxes work and their importance. Public health efforts should invest in campaigns that explain the benefits of SSB taxes and provide information about how tax revenues will be invested, both before and after a tax proposal has passed.

## 1. Introduction

Sugar-sweetened beverages (SSBs) are significant contributors to poor long-term health outcomes, including dental caries, obesity, and cardiometabolic diseases, such as type 2 diabetes [1,2]. SSB consumption is greater among lower-income and racially/ethnically marginalized populations, driving health inequities [3]. SSB taxes, an increasingly popular prevention strategy, have been implemented by local governments in seven US jurisdictions and by 35 nation states around the world [4,5]. A growing body of evidence shows that SSB taxes reduce purchases of SSBs, thus achieving their intended purpose of “nudging” consumers away [6,7]. The demonstrated declines in purchasing are consistent with a priori estimates of price elasticity of demand, suggesting that consumers are responding to higher SSB prices [8,9,10,11].

Strategically framed campaigns for SSB taxes, designed to educate citizens about taxes and why they are important [12,13], may themselves influence people’s behaviors independent of changes in SSB prices. It is known that people’s nutrition knowledge [14] and attitudes and social norms regarding SSBs [15] are associated with their SSB consumption. This highlights the need to consider knowledge and perceptions of SSBs and SSB taxes in designing and evaluating such interventions. Findings from Berkeley, CA demonstrate declines in SSB purchases prior to price changes due to a tax, supporting the hypothesis that tax campaigns themselves can affect SSB consumption, perhaps by affecting knowledge and attitudes [16]. SSB tax campaigns and tax passage could, in fact, also affect perceptions in nearby communities, but studies have not yet examined if people in neighboring untaxed communities are “educated” via pro-tax campaigns.

The extent that tax awareness and perceptions play a role in SSB consumption could also represent a missed opportunity to promote public health. If pro-tax campaign information does not reach the general public or reaches only a subset of the general public, an opportunity for education is missed. Similarly, if the general public is not actually aware that SSB taxes pass, an opportunity to shift social norms has been missed, since the passage of taxes via ballot measure reflects a public consensus that such taxes are important. Additionally, proper framing of SSB tax campaigns has an impact on implementation success once the tax has passed, highlighting the importance of messaging and awareness [17]. Public support for taxes is highest when citizens understand the purpose of the tax, and that tax revenues will be used for the public’s benefit [18]. For example, local leadership and public support for the tax in Berkeley, California was important for its successful implementation [19], whereas in Cook County, Illinois, the primary goal of the tax was to raise revenue to fill a budget deficit, and the tax, lacking sustained public support, was repealed after facing post-enactment litigation [20]. While SSB taxes are top of mind for many public health advocates, it is unclear how aware the general public is of the existence of SSB taxes and their perceptions of such taxes.

Identifying the potential missed opportunities of SSB tax campaigns and passage is particularly important for combatting the SSB industry’s strategy of preemption, which seeks to pass state laws that prohibit the ability of more municipalities to pass new SSB taxes [21]. Preemption was used by the tobacco industry to fight against taxes on tobacco products in the 1980s–1990s and posed a major setback to the tobacco taxation movement at the local level [21]. If the public does not perceive the benefits of SSB taxes, municipalities may be left at greater risk of additional preemption occurring, limiting their ability to pass new SSB taxes. In light of multiple potential missed opportunities, the aims of this research are to understand the level of tax awareness in communities with SSB taxes and in neighboring communities, and to determine if SSB tax awareness and perceptions differ based on sociodemographic characteristics. 

## 2. Materials and Methods

### 2.1. California Bay Area SSB Taxes

**Figure 1:**
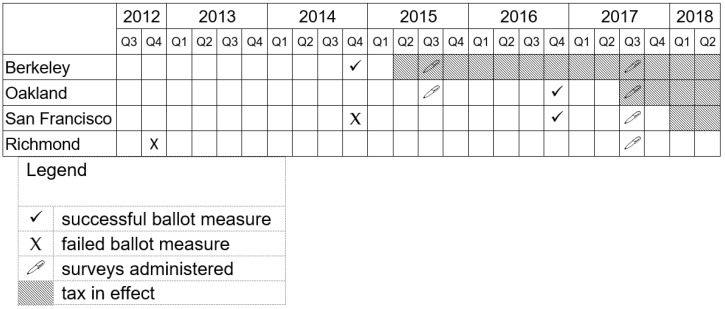
Timeline of SSB ballot measures, tax implementation, and study data collection, 2014–2019.

This study took place against the backdrop of a series of tax ballot measures in multiple cities in the California Bay Area (all new taxes in California must be approved by voters). SSB taxes passed in Berkeley in 2014 and in San Francisco and Oakland in 2016. Ballot measures were unsuccessful in Richmond in 2012 and in San Francisco in 2014 (Figure 1).

### 2.2. Study Design and Participants

Using a serial cross-sectional design, we conducted intercept surveys with residents of Berkeley and Oakland in 2015 and 2017, and in San Francisco and Richmond in 2017, in two demographically diverse neighborhoods in each city. Neighborhood selection and details about survey administration have been described previously [6]. In brief, we identified two neighborhoods in each city with high proportions of low-income, Black, and Latinx residents using 2010 census data. During each year of data collection, trained research assistants conducted intercept surveys with willing participants on busy street corners in each neighborhood and provided reusable bags as incentives for participation.

The analytic sample included 2715 respondents across the 2015 and 2017 data collection periods. The majority of the respondents identified as Black (34%) or Latinx (35%); more respondents identified as White and with college education in Berkeley than in Oakland, San Francisco, and Richmond (Table 1). 

### 2.3. Measures

To assess respondents’ awareness of SSB taxes, we used the following questions: “Think about the election last November. From what you remember, did [city] have an SSB tax on the ballot?”; if a respondent said yes, we also asked, “Do you think it passed?” We define tax “awareness” to be the proportion of residents in a taxed city accurately recalling that a tax had passed. We considered “spillover” to be the proportion incorrectly believing that a tax had passed when one’s city had no tax. Awareness was assessed in Berkeley in 2015 and 2017, and in Oakland and San Francisco in 2017; spillover was assessed in Oakland in 2015 and in Richmond in 2017 (Figure 1).

In 2017, in all four cities, we assessed the perceived benefits of the tax to children’s health and the community (“On a scale of 1 [extremely bad] to 7 [extremely good], how good or bad is the [city] SSB tax for … children’s health? … your community?”) and perceived price of SSBs (“What do you think about the price of sugary drinks in [city]?” from 1 (extremely cheap) to 7 (extremely expensive)). 

Other variables included race/ethnicity, education level, gender, age, and SSB consumption. Education is used as a proxy for socioeconomic status (SES). A validated beverage frequency questionnaire (BFQ-15) [22] was used to determine how often respondents drank regular soda, energy drinks, sports drinks, fruit drinks, and sweetened coffee or tea. We converted all responses to times per day and calculated total SSB consumption by summing the frequencies of individual SSBs. The survey asked between 13 and 17 questions, depending on the year and city. 

### 2.4. Statistical Analyses

All analyses were conducted in Stata/SE 16.1 (StataCorp LLC, College Station, TX, USA). Using mixed effects logistic regression models, we calculated unadjusted and adjusted marginal predicted probabilities of awareness and spillover. We modeled the odds of recalling that a tax had passed, using city and individual-level covariates, such as self-reported SSB consumption, race/ethnicity, education, gender, and age as independent variables, and a random intercept for neighborhood to help us account for observations being clustered within neighborhoods. These models allowed us to determine whether the proportion of respondents who recalled a tax having passed differed by city or respondent characteristics, including SSB consumption, race/ethnicity, and education. 

We calculated adjusted marginal predicted mean perceptions about the benefits of the SSB taxes to the community and children’s health, and the price of SSBs (on a scale from 1 to 7) using mixed effects models, including city and individual level covariates, such as thinking there is a tax, self-reported SSB consumption, race/ethnicity, education, gender, and age as independent variables, with a random intercept for neighborhood. 

## 3. Results

### 3.1. Tax Awareness and Spillover

**Figure 2:**
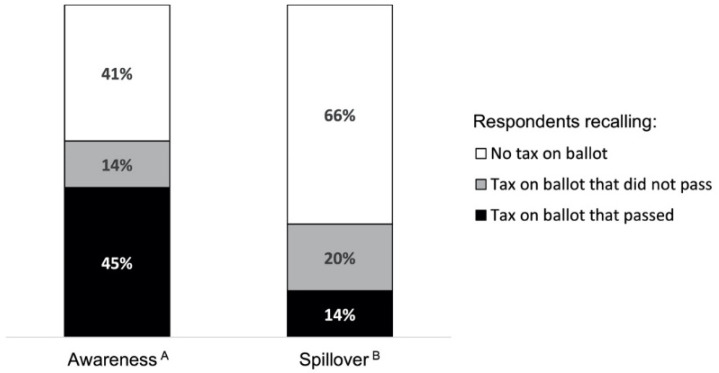
Unadjusted percentage of respondents correctly (Awareness) and incorrectly (Spillover) recalling passage of a tax in prior year. ^A^ In cities where a tax passed via ballot measure in prior year: Berkeley (2015, *N* = 555), Oakland (2017, *N* = 656), and San Francisco (2017, *N* = 593). ^B^ In cities where a tax was not on the ballot in prior year: Oakland (2015, *N* = 472) and Richmond (2017, *N* = 617).

**Figure 3:**
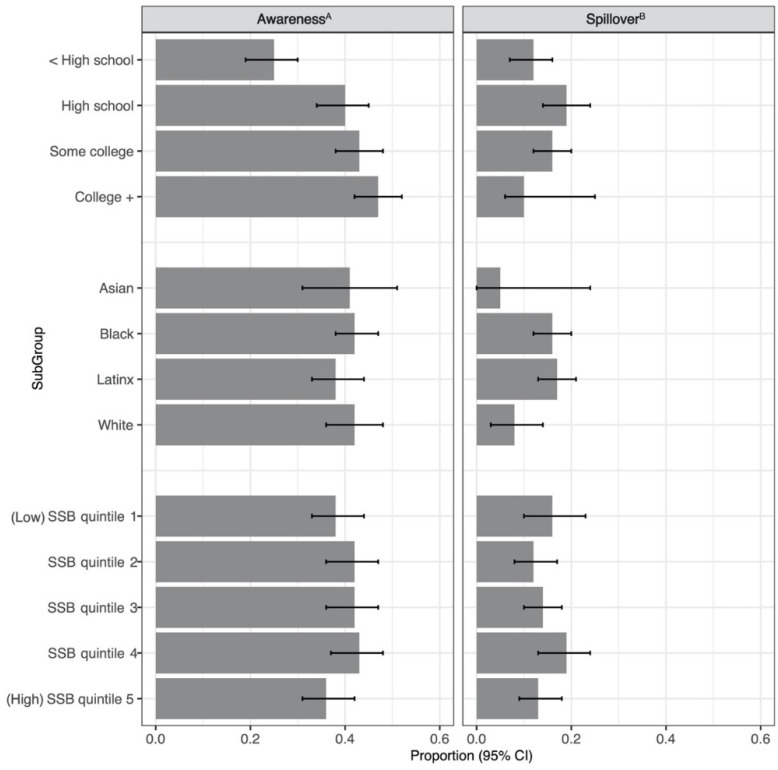
Adjusted proportion of respondents correctly (Awareness) and incorrectly (Spillover) recalling passage of a tax in prior year, by education, race/ethnicity, and quintile of SSB consumption. Adjusted marginal predicted probabilities from logistic mixed-effects model adjusting for quantile of SSB consumption, race/ethnicity, education, gender, and age, with random intercept for neighborhood. ^A^ In cities where a tax passed via ballot measure in prior year: Berkeley (2015), Oakland (2017), and San Francisco (2017), *N* = 1711. ^B^ In cities where a tax was not on the ballot in prior year: Oakland (2015) and Richmond (2017), *N* = 1004.

In the year following successful SSB tax ballot measures, 45% of respondents correctly recalled that an SSB tax had passed in their city (Figure 2), with greater awareness in Berkeley (52%) than in Oakland (39%; *p* < 0.01) or San Francisco (30%; *p* < 0.01). Estimates were similar after adjusting for demographic characteristics and SSB consumption (Table S1). In fully adjusted models, respondents with higher levels of education demonstrated higher levels of tax awareness (*p* < 0.01). There were no differences in the overall effect of race/ethnicity or levels of SSB consumption on tax awareness (Figure 3). Three years after Berkeley’s tax passed (in 2017), 70% of respondents knew there was a tax.

With respect to spillover, 14% of respondents incorrectly thought a tax had passed when it had not (Figure 2). The proportions incorrectly stating that their city had an SSB tax were similar in Richmond (16%) and Oakland (12%; *p* = 0.09). Estimates were similar in fully adjusted models (Table S1). Respondents with a high school degree or some college education were more likely to incorrectly believe there was a tax compared to those with less than a high school degree or college degree (*p* = 0.04). There were no significant differences by race/ethnicity or levels of SSB consumption (Figure 3).

### 3.2. Perceptions of SSB Tax Benefits and Price Changes

**Figure 4:**
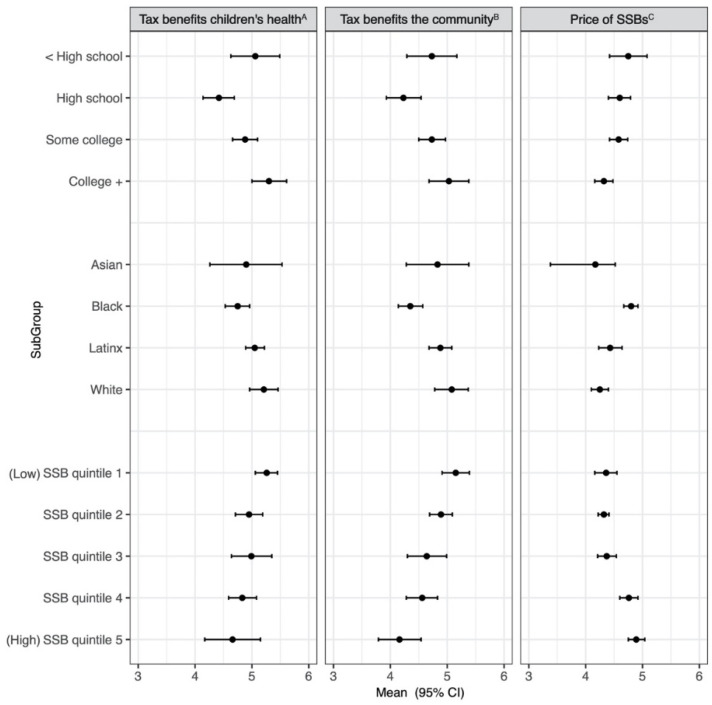
Adjusted mean perceptions about SSB taxes by education, race/ethnicity, and quintiles of SSB consumption, 2017. Adjusted marginal predicted means from mixed-effects models adjust for thinking there is a tax, living in a taxed city, education, quantile of SSB consumption, race/ethnicity, gender, and age, and include random intercept for neighborhood. Results represent adjusted marginal predicted means among full sample of respondents in 2017. ^A^ 1 = extremely bad for children’s health, to 7 = extremely good for children’s health, *N* = 822. ^B^ 1 = extremely bad for the community, to 7 = extremely good for the community, *N* = 819. ^C^ 1 = extremely cheap, to 7 = extremely expensive, *N* = 1166.

Across the sample, on a scale from 1 (“extremely bad”) to 7 (“extremely good”), the unadjusted mean perceived benefit of SSB taxes to the community was 4.5, and to children’s health was 4.7 (Figure S1). In fully adjusted models, there were significant differences in perceived benefits to both children and the community by education (*p* < 0.01), race/ethnicity (*p* < 0.01), and SSB consumption (*p* < 0.01) (Table S2). On average, those with a high school education perceived fewer benefits than others, and Black respondents perceived fewer benefits than Latinx and White respondents (Figure 4). As SSB consumption increased, respondents perceived declining benefits (Figure 4). Believing that one’s city had a tax was not associated with perceptions about benefits, regardless of whether respondents were correct in their belief. 

On a scale from 1 (“extremely cheap”) to 7 (“extremely expensive”), the overall mean perceived price of SSBs was 4.5 (Figure S1). In adjusted models, believing there was a tax was associated with perceiving SSBs to be more expensive (*p* < 0.01). On average, respondents who thought there was an SSB tax perceived the price to be higher than those who did not think there was a tax (4.7 (95% CI: 4.5, 4.8) versus 4.3 (95% CI: 4.2, 4.4), respectively), adjusting for the actual presence of a tax. The effects of education (*p* < 0.01), race/ethnicity (*p* < 0.01), and SSB consumption (*p* < 0.01) on mean perceived price were also statistically significant (Table S2): as education level increased, respondents perceived SSBs to be less expensive. Black respondents perceived a higher price of SSBs than Asian, Latinx, and White respondents, and more frequent SSB consumers were more likely to believe that SSBs were costly (Figure 4). 

## 4. Discussion

This study examined SSB tax awareness and perceptions among citizens in four California Bay Area cities, some with a municipal SSB tax and others without. Overall, we found rather limited awareness of SSB taxes, even though the Bay Area experienced six different SSB tax campaigns between 2012 and 2016 [5]. In the year following successful SSB tax ballot propositions in three Bay Area cities, less than half of the respondents in low-income neighborhoods were aware that an SSB tax had passed, although it is possible that awareness of taxes increases with time. In Berkeley, the first city to tax SSBs, awareness of the tax increased from 52% to 70% over two years. In nearby Bay Area cities without an SSB tax, nearly 15% of respondents incorrectly believed that their city had a tax. Perceived benefits of SSB taxes to the community and to children’s health were moderate and, like correct recall of an SSB tax, were higher among respondents with higher education levels.

Our findings suggest an uneven awareness of taxes among residents by educational attainment, with the least awareness among those with the lowest levels of education. This is consistent with research showing that populations with lower education have greater barriers to accessing health-related information [23,24]. Despite being less aware of the tax, people with lower levels of education are more sensitive to price changes. Prior research has shown that people do, in fact, notice price increases, but they do not necessarily attribute the price increase to the tax [25]. Disparities in awareness of SSB taxes could affect the taxes’ implementation and ultimate success. A body of literature has demonstrated that diffusion of new information and uptake of health messages is lower in lower-income, socially disenfranchised communities [11,23,24]. If tax awareness is lower in lower-income communities, responses to taxes might be blunted, which could widen the current gap in consumption by SES. Lower-income people are found to have poorer nutrition knowledge [11] and have even more to gain in terms of increasing knowledge of the health effects of SSB consumption by learning about SSB taxes. Thus, differential awareness of taxes would represent a missed opportunity. Future campaigns for new SSB taxes should be intentional in their messaging and in engaging lower-SES communities.

Tax awareness was highest in Berkeley. Berkeley’s tax, the first in the nation, passed with 76% of the vote, despite counter-advertising from the soda industry of nearly USD 2.4 million, or about USD 30 for each registered voter in Berkeley [19,26]. Leading up to the ballot measure, the soda industry blanketed the small city with advertisements in public transportation stations, local newspapers, and public spaces around town [27], while supporters of the tax handed out 2000 lawn signs and went door to door to talk to voters [28]. The grassroots, “Berkeley vs. Big Soda” campaign may provide guidance on salient messaging for additional campaigns and is an example of how SSB tax campaigns can serve as opportunities to educate the public about SSBs. 

Our findings from the Bay Area suggest that information about the benefits or revenue allocation of taxes may not be reaching the public, particularly residents of lower-SES communities. This is ironic because a key goal of SSB taxes is to address health disparities by generating revenue to fund community chronic disease prevention efforts in under-resourced communities [29], and the majority of revenue allocations from municipal SSB taxes in the US are directed toward low-SES communities [30]. In particular, respondents with lower levels of education, and those who identified as Black, were less likely to believe that SSB taxes benefit the community and children’s health compared to those with higher levels of education and those who identified as Asian, Latinx, or White. This could be a reflection of longstanding barriers to accessing health information for populations with lower education [23,24]. In Bay Area cities with taxes, there had been almost a complete lack of SSB tax revenue being used for ongoing communication about the tax and where its revenues were being allocated [30].

Interestingly, regardless of whether one lived in a taxed city, believing there was a tax was associated with thinking SSB prices were too high, even after controlling for SSB consumption. However, prior studies of the Bay Area document that SSB prices increased more in cities with a tax than in neighboring cities [31,32]. This “spillover” of perceptions of high prices could have a beneficial effect, helping nudge people away from SSBs in untaxed cities. 

This study has several limitations. Surveys were collected using a convenience sample, which may limit the generalizability of our findings. We may have unmeasured confounding affecting our estimates. Findings from California’s Bay Area may not generalize to other cities. Surveys were collected the year following the ballot measure in all taxed cities, but prior to implementation of the tax in San Francisco. Differences in the timing of the surveys with respect to tax implementation may have affected respondents’ awareness and perceptions of the tax. We only surveyed about awareness of taxes the year following their passage and perceptions of the benefits and price in 2017; thus, we lack longer-term data.

## 5. Conclusions

We find that awareness of SSB taxes is low overall and that perceptions about taxes’ benefits vary by educational attainment, reflecting a missed opportunity to educate citizens about how SSB taxes work and why they are important. Public health efforts should invest in campaigns that explain the benefits of SSB taxes and provide information about how tax revenues will be invested, both before and after a tax proposal has passed. Proponents of SSB taxes hope that higher prices nudge consumers to purchase fewer SSBs [33]. More research is needed to understand the longer-term implications of SSB tax social marketing on tax implementation success, SSB consumption, and public health impacts.

## Figures and Tables

**Table 1:** Respondent characteristics by city, 2015 and 2017.

*n* (%)	Total	Berkeley ^A^	Oakland ^A^	San Francisco ^A^	Richmond ^A^
	*N* = 2715	*N* = 524	*N* = 1042	*N* = 564	*N* = 585
Year of interview					
2015	943 (35%)	524 (100%)	419 (40%)	0 (0%)	0 (0%)
2017	1772 (65%)	0 (0%)	623 (60%)	564 (100%)	585 (100%)
Race/ethnicity					
Asian	151 (6%)	30 (6%)	37 (4%)	42 (7%)	42 (7%)
Black	934 (34%)	164 (31%)	433 (42%)	131 (23%)	206 (35%)
Latinx	957 (35%)	110 (21%)	411 (39%)	235 (42%)	201 (34%)
Other	215 (8%)	56 (11%)	77 (7%)	42 (7%)	40 (7%)
White	458 (17%)	164 (31%)	84 (8%)	114 (20%)	96 (16%)
Education					
<High school	484 (18%)	52 (10%)	254 (24%)	120 (21%)	58 (10%)
High school	665 (24%)	99 (19%)	287 (28%)	120 (21%)	159 (27%)
Some college	782 (29%)	138 (26%)	314 (30%)	133 (24%)	197 (34%)
College grad +	784 (29%)	235 (45%)	187 (18%)	191 (34%)	171 (29%)
Gender					
Female	1564 (58%)	278 (53%)	668 (64%)	302 (54%)	316 (54%)
Male	1151 (42%)	246 (47%)	374 (36%)	262 (46%)	269 (46%)
Age					
18–29	906 (25%)	215 (24%)	280 (27%)	224 (20%)	187 (32%)
30–39	720 (20%)	147 (16%)	186 (18%)	270 (24%)	117 (20%)
40–49	566 (15%)	133 (15%)	182 (17%)	181 (16%)	70 (12%)
50–59	704 (19%)	174 (19%)	197 (19%)	238 (21%)	95 (16%)
≥60	788 (21%)	236 (26%)	202 (19%)	234 (20%)	116 (20%)
SSB consumption (quintile)					
1 (low)	515 (19%)	153 (29%)	144 (14%)	117 (21%)	101 (17%)
2	525 (19%)	104 (20%)	189 (18%)	120 (21%)	112 (19%)
3	585 (22%)	90 (17%)	216 (21%)	139 (25%)	140 (24%)
4	585 (22%)	97 (19%)	249 (24%)	118 (21%)	121 (21%)
5 (high)	505 (19%)	80 (15%)	244 (23%)	70 (12%)	111 (19%)

^A^ Berkeley passed an SSB tax in 2014 and Oakland and San Francisco in 2016; Richmond has not passed a tax.

## Data Availability

The data presented in this study are available on request from the corresponding author. The data are not publicly available due to ongoing investigations.

## Supplementary Materials

The following are available online at https://www.mdpi.com/article/10.3390/ijerph18094607/s1: **Figure S1**: Unadjusted proportion of responses for perceptions about SSB taxes; Table S1: Adjusted proportion of respondents correctly (Awareness) and incorrectly (Spillover) recalling passage of a tax in prior year, by city, education, race/ethnicity, and quintile of SSB consumption; Table S2: Adjusted mean perceptions about SSB taxes by city, education, race/ethnicity, and quintiles of SSB consumption, 2017.
