# Supplementary material for: Missed Opportunities: The Need to Promote Public Knowledge and Awareness of Sugar-Sweetened Beverage Taxes
Source: Int J Environ Res Public Health. 2021 Apr 27;18(9):4607. doi: 10.3390/ijerph18094607 (PMC8123585; doi:10.3390/ijerph18094607)
Supplement: Supplementary file 1 [file ijerph-18-04607-s001.zip › ijerph-1142917 Supplementary final.pdf]

Supplemental Materials

Figure S1: Unadjusted proportion of responses for perceptions about SSB taxes

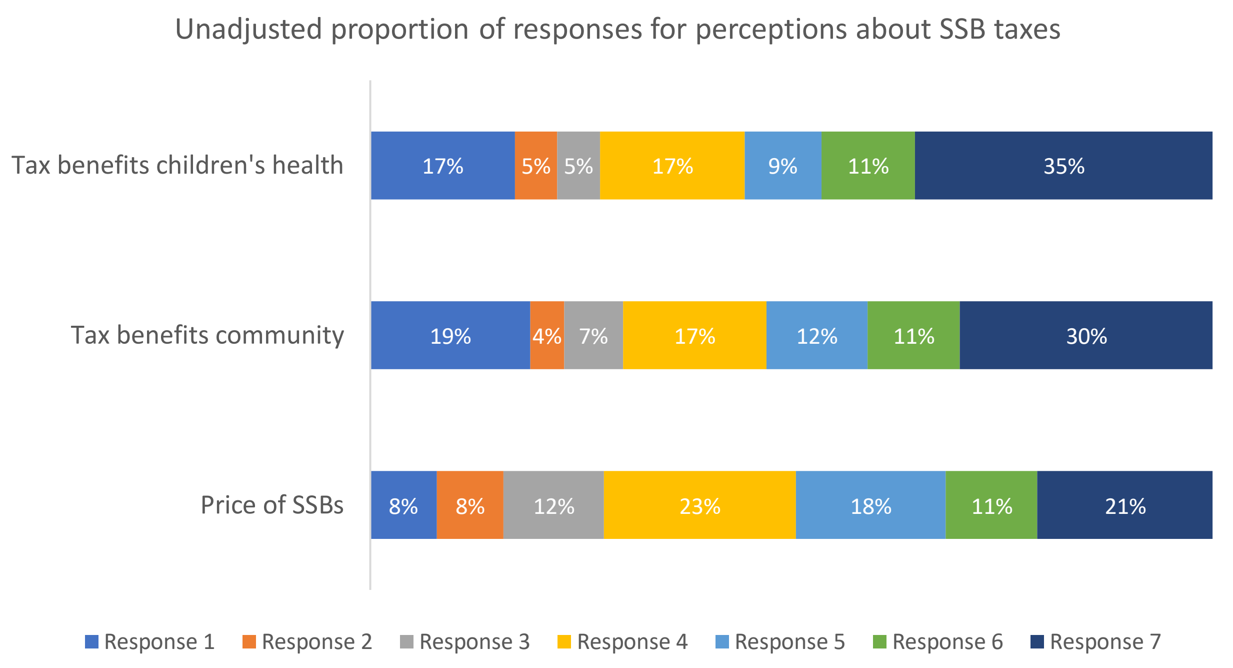

Unadjusted proportions for thinking the tax benefits children's health (N=822), where responses range from 1=Extremely bad for children's health, to 7=Extremely food for children's health (mean=4.7, standard deviation=2.3)

Unadjusted proportions for thinking the tax benefits the community (N=819), where responses range from 1=Extremely bad for the community, to 7=Extremely food for the community (mean=4.5, standard deviation=2.2)

Unadjusted proportions for the price of SSBs (N=1166), where responses range from 1=Extremely cheap, to 7=Extremely expensive (mean=4.5, standard deviation=1.8)

Table S1: Adjusted proportion of respondents correctly (Awareness) and incorrectly (Spillover) recalling passage of a tax in prior year, by city, education, race/ethnicity, and quintile of SSB consumption

	Awareness[A]	Spillover[B]
City		
Berkeley	0.50 (0.43, 0.57)	N/A
Oakland	0.41 (0.36, 0.46)	0.11 (0.09, 0.14)
San Francisco	0.31 (0.26, 0.35)	N/A
Richmond	N/A	0.17 (0.14, 0.21)
Education		
< High school	0.25 (0.19, 0.30)	0.12 (0.07, 0.16)
High school	0.40 (0.34, 0.45)	0.19 (0.14, 0.24)
Some college	0.43 (0.38, 0.48)	0.16 (0.12, 0.20)
College +	0.47 (0.42, 0.52)	0.10 (0.06, 0.25)
Race/ethnicity		
Asian	0.41 (0.31, 0.51)	0.05 (0, 0.24)
Black	0.42 (0.38, 0.47)	0.16 (0.12, 0.20)
Latinx	0.38 (0.33, 0.44)	0.17 (0.13, 0.21)
White	0.42 (0.36, 0.48)	0.08 (0.03, 0.14)
SSB consumption		
SSB quintile 1 (low)	0.38 (0.33, 0.44)	0.16 (0.10, 0.23)
SSB quintile 2	0.42 (0.36, 0.47)	0.12 (0.08, 0.17)
SSB quintile 3	0.42 (0.36, 0.47)	0.14 (0.10, 0.18)
SSB quintile 4	0.43 (0.37, 0.48)	0.19 (0.13, 0.24)
SSB quintile 5 (high)	0.36 (0.31, 0.42)	0.13 (0.09, 0.18)

[A] In cities where a tax passed via ballot measure in prior year: Berkeley (2015), Oakland (2017), and San Francisco (2017)
[B] In cities where a tax was not on the ballot in prior year: Oakland (2015) and Richmond (2017)

Table S2: Adjusted mean perceptions about SSB taxes by city, education, race/ethnicity, and quintiles of SSB consumption, 2017

	Tax benefits children's health[A]	Tax benefits the community[B]	Tax increases price of SSBs[C]
City			
Berkeley	5.12 (5.05, 5.19)	4.86 (4.82, 4.90)	4.65 (4.57, 4.72)
Oakland	4.44 (4.35, 4.54)	4.49 (4.37, 4.60)	4.54 (4.37, 4.72)
San Francisco	5.00 (4.87, 5.14)	4.70 (4.59, 4.82)	4.44 (4.40, 4.49)
Richmond	5.20 (5.13, 5.28)	4.87 (4.61, 5.13)	4.43 (4.35, 4.51)
Education			
< High school	5.06 (4.63, 5.49)	4.73 (4.29, 5.17)	4.75 (4.42, 5.08)
High school	4.42 (4.14, 4.69)	4.23 (3.93, 4.54)	4.60 (4.40, 4.79)
Some college	4.88 (4.66, 5.1)	4.73 (4.50, 4.97)	4.58 (4.42, 4.74)
College +	5.30 (5.00, 5.61)	5.03 (4.68, 5.38)	4.32 (4.16, 4.48)
Race/ethnicity			
Asian	4.90 (4.26, 5.53)	4.83 (4.28, 5.38)	4.17 (3.38, 4.52)
Black	4.75 (4.53, 4.96)	4.35 (4.14, 4.57)	4.80 (4.67, 4.92)
Latinx	5.05 (4.89, 5.22)	4.88 (4.68, 5.08)	4.43 (4.23, 4.64)
White	5.21 (4.96, 5.46)	5.08 (4.78, 5.37)	4.25 (4.10, 4.40)
SSB consumption			
SSB quintile 1 (low)	5.26 (5.06, 5.45)	5.15 (4.91, 5.39)	4.36 (4.16, 4.55)
SSB quintile 2	4.95 (4.71, 5.19)	4.89 (4.69, 5.09)	4.32 (4.22, 4.41)
SSB quintile 3	4.99 (4.64, 5.35)	4.64 (4.30, 4.99)	4.37 (4.21, 4.54)
SSB quintile 4	4.83 (4.59, 5.08)	4.56 (4.28, 4.83)	4.76 (4.60, 4.92)
SSB quintile 5 (high)	4.66 (4.17, 5.15)	4.16 (3.79, 4.54)	4.89 (4.75, 5.04)

[A] 1=Extremely bad for children's health, to 7=Extremely good for children's health, N=822
[B] 1=Extremely bad for the community, to 7=Extremely good for the community, N=819
[C] 1=Extremely cheap, to 7=Extremely expensive, N=1166
